# Incidence of Medication Discrepancies and Its Predicting Factors in Emergency Department

**Published:** 2017-08

**Authors:** Morvarid ZARIF-YEGANEH, Mansoor RASTEGARPANAH, Gholamreza GARMAROUDI, Molouk HADJIBABAIE, Hojjat SHEIKH MOTAHAR VAHEDI

**Affiliations:** 1. Faculty of Pharmacy, Tehran University of Medical Sciences, Tehran, Iran; 2. Dept. of Clinical Pharmacy, Faculty of Pharmacy, Tehran University of Medical Sciences, Tehran, Iran; 3. Dept. of Health Promotion and Education, School of Public Health, Tehran University of Medical Sciences, Tehran, Iran; 4. Research Center for Rational Use of Drugs, Dept. of Clinical Pharmacy, Faculty of Pharmacy, Tehran University of Medical Sciences, Tehran, Iran; 5. Dept. of Emergency Medicine, Shariati Hospital, Tehran University of Medical Sciences, Tehran, Iran

**Keywords:** Medication discrepancies, Medication reconciliation, Emergency department

## Abstract

**Background:**

This study was conducted to evaluate the incidence of medication discrepancies and its related factors using medication reconciliation method in patients admitted to the emergency department of Tehran University of Medical Sciences hospitals.

**Methods:**

In this cross-sectional study, 200 adult patients with at least one chronic disease that used two regular prescription medications were included in 2015. After 24 h of admission, demographic data and patient’s home medications were collected. Medication discrepancies were assessed through comparison of a best possible medication history list with the physician’s orders.

**Results:**

Out of 200 patients (mean age, 61.5 yr; 86 males, 114 women), 77.5% of patients had one or more medication discrepancies. The most common discrepancies were medication omission (35.49%), change (14.22%) and substitution (10.97%), respectively. The relationship between number of comorbid conditions (*P*=0.025), regular home medications (*P*=<0.001), high-risk medications (*P*=0.032), medications pharmacological classes (*P*=<0.001) and medication discrepancies were statistically significant. Cardiovascular drugs compared to other medications classes showed the highest discrepancies (36.2%). Multiple logistic regression showed that the drug groups, including anti-infective for systemic use (OR=8.43; 95%CI 2.5–28.2; *P*=0.001), Antineoplastic and Immuno-modulator Agents (OR=0.49; 95%CI 0.27–0.87; *P*=0.016), Blood and Blood-Forming Organs (OR=0.33; 95%CI 0.21–0.52; *P*<0.001), Muscular-Skeletal System (OR=2.4; 95%CI 1.13–5.1; *P*=0.022), Nervous-System (OR=2.75; 95%CI 1.7–4.4; *P*<0.001), Respiratory-System (OR=0.38; 95%CI 0.22–0.67; *P*=0.001) were associated with the drug discrepancy.

**Conclusion:**

A medication discrepancy occurs commonly at hospital emergency department. Understanding the type and frequency of discrepancies with using structured medication reconciliation process can help clinicians to prevent them.

## Introduction

In all societies, medications are important because of their capacity to treat and prevent disease and to support Public Health Programs (PHPs). All medications carry some risk of harm, therefore, it is important to monitor their effects (both intended and unintended). Despite pharmacovigilance improvement, the adverse drug reactions burden on public health because of unintended medication errors (traditionally referred to as ADRs) remains significant (1).

The NHS (National Health Service) Commissioning Board (UK) identified medication errors as a major public health burden; however, many such errors can result in severe patient injury or death but these errors are preventable. Medication errors occur on a daily basis in all healthcare settings and processes, from ordering, dispensing, administering, and monitoring (2, 3). One of the effective factors on the occurrence of medication errors is medication discrepancies, although one discrepancy does not necessarily mean an error (4–6).

Medication discrepancies are any differences between home medications and documented drug regimens across different parts of care, for example admission, transfer, and discharge. Some are intended adjustment (for therapeutic goals), but others are unintentional and clinically inessential (7). Due to poor communication and unintentional information gap, these discrepancies put patients at risk for medication errors (4). In fact, most discrepancies are due to not accommodating past medication to the patient’s newly recognized condition, or because the interventions performed, could interfere with their usual medications (8).

Unintended medication discrepancies that represent errors are common at the time of hospital admission, up to 67% of patients admitted to the hospital have unintended medication discrepancies, and these discrepancies remain common at discharge (4, 9–11).

Medication discrepancies are common at admission or discharge stage, with nearly one-third of these had potential to cause patient harm (potential adverse drug events, PADEs) and contribute to adverse drug events (ADEs) (4). In the post-discharge period, these errors may result in drug interactions, additional costs and inaccurate treatment (5, 11). ADEs related with medication discrepancies can lead to emergency room visits, hospital readmissions, and employment of other health care resources (12). On the other hand, a research has shown that the PADEs are associated with 46%–56% of all medication errors and 1 of 5 injuries or deaths and a result of poorly designed systems (13).

One strategy to reduce medication discrepancies and ADEs is medication reconciliation (13). Using multidisciplinary approach and cooperation among physician, pharmacist and nurse can lead to accomplishment of advanced medication reconciliation and reduction of medication discrepancies (4).

Medication reconciliation is a formal and accurate process of collecting best possible medication history list and information, including drug name, frequency, dosage and route of administration and comparing that list and information with the physician’s orders, with the intention of providing correct medications to the patient at all transition points (4).

Since the patient safety may be compromised during all stages of caring, because of medication error, incomplete information transfer, and insufficiency of coordination with the next provider of care, the Joint Commission on Accreditation of Healthcare Organizations (JCAHO) acknowledged that reconciliation errors could assure the safety of drugs use and receiving the necessary drugs for the new situation with patients. Therefore, recommended hospitals to develop a system of reconciliation for obtaining patients’ complete pharmaco-therapeutic records (8).

By taking the benefits of medication reconciliation, this method has been approved by patient safety organizations in some countries (11) but in hospitals of Islamic Republic of Iran until now, this method has not been used, so that research in this area is necessary.

The first aim of this study was detecting medication discrepancies between home medications and medications ordered in an emergency department with using of medication reconciliation method and determining the incidence of medication discrepancies. The second was identifying which pharmacological categories is more involved in occurred medication discrepancies and which factors are effective on these discrepancies.

## Methods

This cross-sectional study was conducted in the Emergency Department (ED) at Shariati Hospital, which is a tertiary care hospital and academic medical center located in Tehran, Iran. It has 834 beds and ED annual admission of this hospital is 21000. During 8 months, from Jan to Aug 2015, all patients admitted to this emergency department unit with using of medical records were investigated. However, for weekend and holiday admissions this investigation was conducted on the first working day after the admissions.

Each medical record was screened, and patients with at least two regular prescription medications before admission and 24 h had passed since their admission were entered into this study.

The following patients: who were unable to communicate and did not have a caregiver to could be interviewed, patients who were under 18 yr old, and patients not recorded any chronic diseases were excluded from study. We also excluded patients who had not an ability to swallow the prescription medications.

At this hospital, clinical pharmacists covered some teaching units and not involved in the services of pharmaceutical care in emergency department. To produce a verified medication list, a pharmacy student under the supervision of a clinical pharmacist reviewed each medical record to collect physician-ordered admission medications. For preparing a complete medication history database, information about all prescription and nonprescription medications, herbal and vitamin supplements also obtained from a comprehensive interview with patients or their caregivers, inspection of prescriptions container and consulting with family members. In addition, demographic information, chronic diseases, history of allergy and admission services for all patients were collected.

In this study medication, discrepancy defined as any differences between home medications and admission medication orders.

The type classified discrepancies:

1. Omission of medications,
2. Change in drug dose,
3. Frequency or route of administration,
4. Replace or substitution of an agent within the same pharmacologic effect.

Finally, all these type of discrepancies with using of reconciliation method, also relationship between determined discrepancies and other variables such as: demographic data, pharmacological categories (grouped with using of WHO ATC codes), wards, admission time, admission services, smoking status, allergy status, chronic condition with applying of statistically analysis methods were evaluated.

Data were entered into the Stata (ver. 12) for analysis. Descriptive statistics were used to describe the study variables and for normally distributed continuous variables. Binomial logistic regression was performed for bivariate associations between baseline variables and discrepancies and results are presented as odd ratio (OR), %95 CI and *P*-value. Finally, variables that showed statistical significance with response variable (discrepancies) entered into a multivariable logistic regression model (adjusted).

Patients or family members provided written informed consent and Tehran University of Medical Sciences Ethics Board approved the study protocol.

## Results

During the first 8 months, 2047 patients were screened and of these, 200 were enrolled. Other patients for the following reasons were excluded: 24 h had not passed of their admission (n=1393), using fewer than 2 medications (n=358), could not give informed consent and refused to participate (n=17), or were aged <18 yr (n=6). An additional 73 patients were excluded because of inability to communicate, with no available family members. For completing medication history, medical records of all 200 participants were investigated. Furthermore, other main information sources, including: interview with patients (24 (12%)), interview with caregivers (18 (9%)), interview with patients and using of medication bottles (24 (12%)), interview with caregivers and using of medication bottles (71 (35.5%)), interview with patients and caregivers also using of medication bottles (44 (22%) and other (19 (9.5%)) were used. The characteristics of the 200 patients in the study population are summarized in Table 1.

**Table 1:** Patients' demographic and clinical characteristics

**Variables**	**Discrepancy**		**Total**

	**No, n (%)**	**Yes, n (%)**	
**Age(yr)**			
18–29	2 (25)	6 (75)	8 (100)
30–39	8 (38.10)	13 (56.25)	16 (100)
40–49	7 (43.75)	9 (56.25)	16 (100)
50–59	8 (21.62)	29 (78.37)	37 (100)
>=60	20 (16.95)	98 (83. 05)	118 (100)
**Sex**			
male	19 (22.09)	67 (77.61)	86 (100)
Female	26 (22.81)	88 (77.19)	114 (100)
**Education Level**			
Illiterate	13 (20.97)	46 (79.03)	62 (100)
Did not graduate from high school	20 (24.66)	61 (75.31)	81 (100)
High school graduate	8 (21.05)	30 (78.95)	38 (100)
At least some college/university/trade school	4 (21.05)	15 (78.95)	19 (100)
**Admission time**			
Morning(7 AM–1 PM)	19 (24.68)	58 (75.32)	77 (100)
Evening(1 PM–7 Night)	18 (20.45)	70 (79.55)	88 (100)
Night(7 Night–7 AM)	8 (22.86)	27 (77.14)	35 (100)
**Admission services**			
Internal	7 (19.44)	29 (80.56)	36 (100)
Emergency	5 (9.80)	46 (90.20)	51 (100)
Heart	5 (25.00)	15 (75.00)	20 (100)
Nephrology	7 (46.67)	8 (53.33)	15 (100)
Neurology	7 (43.75)	9 (56.35)	16 (100)
Lung	5 (35.71)	6 (64.29)	14 (100)
Gastro-intestinal (GI)	3 (23.08)	10 (76.92)	13 (100)
Rheumatology	5 (35.71)	9 (64.29)	14 (100)
Surgery	1 (10.00)	9 (90.00)	10 (100)
Endocrinology	0 (0.00)	5 (100.00)	5 (100)
Orthopedic	0 (0.00)	2 (100.00)	2 (100)
Urology	0 (0.00)	2 (100.00)	2 (100)
Hematology	0 (0.00)	1 (100.00)	1 (100)
Neurosurgery	0 (0.00)	1 (100.00)	1 (100)
**Smoking status**			
No	41 (23.56)	133 (76.44)	174 (100)
Yes	4 (15.38)	22 (84.62)	26 (100)
**Allergy status**			
Drug allergy	6 (18.75)	26 (81.25)	32 (100)
Chemical allergy	1 (50.00)	1 (50.00)	2 (100)
Non	38 (22.89)	128 (77.11)	166 (100)
**Chronic condition**			
Hypertension (HTN)	26 (23.01)	87 (76.99)	113 (100)
Diabetes Mellitus (DM)	11 (15.71)	59 (84.29)	70 (100)
Dyslipidemia (DLP)	10 (18.52)	44 (81.48)	54 (100)
Cerebrovascular Accident (CVA)	3 (16.67)	15 (83.33)	18 (100)
End Stage Renal Disease (ESRD)	6 (22.22)	21 (77.78)	27 (100)
Congestive Heart Failure (CHF)	3 (15.79)	16 (84.21)	19 (100)
Ischemic Heart Disease (IHD)	11 (15.71)	59 (84.29)	70 (100)
Seizure	0 (0.00)	5 (100.00)	5 (100)
Hypothyroidism	3 (15.79)	16 (84.21)	19 (100)
Inflammatory Bowel Disease (IBD)	3 (30.00)	7 (70.00)	10 (100)

Of the 200 patients, 128 patients (64%) reported taking less and equal to 7 drugs and 113 patients (56.5%) had at least 1 high-risk medication (Warfarin, digoxin, insulin, oral anti-diabetic medications, phenytoin, carbamazepine, lithium carbonate, low molecular weight heparin, methotrexate, cyclosporine, azathioprine, mycopheno-late mofetil, thyroid hormones, theophylline, narcotics, opiates and chemotherapy drugs). However, the mean of home prescription medications use were 6.79 (SD 2.95) drugs per patient. One hundred fifty-five patients (77.5%) had greater and equal to one discrepancy and among the 1358 medication discrepancies, 824 (60.68%) were determined to be unintentional. The most common unintentional error was omission of a regularly used medication, and most discrepancies happened in the cardiovascular drugs (36.17%) and Nervous system drugs (18.45%). Other types of unintentional medication discrepancies with number of unintentional medication discrepancies in medication groups are summarized in Table 2.

**Table 2:** Number of unintentional medication discrepancies by type

**Type of discrepancies**	**Medication discrepancies; number (%)**	**Medication Groups (grouped by ATC codes), number of discrepancies**										
		A	J	L	B	C	G	M	N	R	H	V
Omission	482(58.5%)	32 (26.67)	10 (24.39)	13 (48.15)	25 (50.00)	167 (56.04)	14 (100)	31 (79.49)	138 (90.79)	27 (93.10)	18 (38.30)	3 (100)
Substitution	149(18.1%)	74 (61.67)	23 (56.10)	0 (0.00)	5 (10.00)	21 (7.05)	0 (0.00)	8 (20.51)	4 (2.63)	1 (3.45)	13 (27.66)	0 (0.00)
Change	193(23.4%)	14 (11.67)	8 (16.51)	14 (51.85)	20 (40.00)	110 (36.91)	0 (0.00)	0 (0.00)	10 (6.58)	1 (3.45)	16 (34.04)	0 (0.00)
Total	824(100%)	120 (100)	41 (100)	27 (100)	50 (100)	298 (100)	14 (100)	39 (100)	152 (100)	29 (100)	47 (100)	3 (100)

ATC codes: A, Alimentary Tract and Metabolism; J, Antiinfectives for Systemic Use; L, Antineoplastic and Immunomodulating Agents; B, Blood and Blood forming organs; C, Cardiovascular System; G, Genitourinary System and sex hormones; M, Musculo-Skeletal System; N, Nervous System; R, Respiratory System; H, Systemic Hormonal Preparations; V, Various

ATC, Anatomic Therapeutic Chemical classification

Multiple logistic regression models (adjusted) showed that medication groups, including Cardiovascular System, Genitourinary System and sex hormones, Systemic Hormonal Preparations and Various (other used drugs by patients) were not correlated with discrepancies (Table 3).

**Table 3:** Affected medication groups on medication discrepancies

**Discrepancies**	**Number (%)**	**Medications Groups**	**Odds ratio**	***P*-value**	**95% CI**
Intentional	534(39.32%)		Reference		
		Alimentary Tract and Metabolism		Reference	
		Antiinfectives for Systemic Use	8.42	0.001	2.51–28.19
		Antineoplastic and Immunomodulating Agents;	0.48	0.016	0.27–0.87
		Blood and Blood forming organs	0.33	<0.001	0.21–0.52
Unintentional	824(60.68%)	Cardiovascular System	0.89	0.528	0.63–1.25
		Genitourinary System and sex hormones	2.15	0.189	0.68–6.8
		Musculo-Skeletal System	2.4	0.022	1.13–5.1
		Nervous System	2.7	<0.001	1.72–4.41
		Respiratory System	0.38	0.001	0.22–0.67
		Systemic Hormonal Preparations	1.2	0.517	0.68–2.13
		Various	0.92	0.933	0.15–5.55

However, there was no relationship between using of high-risk drugs (OR=2.00; 95%CI 0.92–4.36; *P*= 0.079) that the patient was taking before admission with happened discrepancies. Although relationship between number of high-risk drugs (OR=1.71; 95%CI 1.05–2.77; *P*= 0.028) that the patient was taking before admission and total number of regular home medications used by patients (OR=1.67; 95%CI 1.35–2.07; *P*=<0.001) with discrepancies was statistically significant. The association between discrepancies and other potential variables was investigated (Table 4). No significant associations were observed between discrepancies and age, sex, education level, admission time, admission wards, allergy status, information sources, patient’s chronic conditions, including: Hypertension, diabetes mellitus, dyslipidemia, cerebrovascular accident, end stage renal disease, congestive heart failure, ischemic heart disease, seizure, hypothyroidism and inflammatory bowel disease. Correlation between medication discrepancies and number of comorbid conditions was statistically significant (Table 4).

**Table 4:** Association between selected variables and discrepancies

**Variables**	**Odds ratio**	***P*-value**	**(95% CI)**
Age(yr)	1.01	0.258	0.99–1.03
Sex	1.24	0.608	0.54–2.85
Education Level	1.28	0.152	0.91–1.80
Admission time	1.01	0.756	0.91–1.12
Admission services	1.05	0.250	0.96–1.15
Allergy status	0.91	0.845	0.39–2.13
Hypertension (HTN)	0.54	0.226	0.20–1.45
Diabetes Mellitus (DM)	1.85	0.240	0.66–5.18
Dyslipidemia (DLP)	1.70	0.306	0.61–4.74
Cerebrovascular Accident (CVA)	0.79	0.753	0.19–3.23
End Stage Renal Disease (ESRD)	1.06	0.918	0.31–3.56
Congestive Heart Failure (CHF)	1.18	0.815	0.28–4.91
Ischemic Heart Disease (IHD)	1.36	0.552	0.49–3.75
Seizure	1	-	-
Hypothyroidism	3.11	0.291	0.37–25.57
Inflammatory Bowel Disease (IBD)	1.88	0.584	0.19–18.36
Information sources	1.08	0.263	0.93–1.26
Number of comorbid conditions	1.41	0.033	1.02–1.94

CI, Confidence Interval

## Discussion

In our study population, 77.5% experienced unintentional medication discrepancies. The most common type of discrepancies was drug omission. We found the relationship between number of comorbid conditions, number of regular home medications, number of high-risk medications, medications pharmacological classes and medication discrepancies.

In other countries, hospitals are at different phases of implementation of medication reconciliation (14). Only one Iranian studies investigated the medication reconciliation effects on medication discrepancies at admission time (15). Medication discrepancies frequently occur at transferring of patients between different wards and especially at admission time (16). In our study, average of unintended discrepancies was 4.12 per patient. This amount of unintended discrepancies in compared with other studies is very high (11, 17). Different explanations of medication discrepancies and changeability in methods of data collection could explain the variations between investigations and make it difficult to correlate rates of unintended medication discrepancies among studies. Most of unintended medication discrepancies were erroneous omissions and wrong doses of drugs (5, 18–20). According to our results and some other studies omission was the most frequent unintended medication discrepancy.

Medication classes were proposed before admission in compare to number of regular home medications may be the most important predictor of unintended medication discrepancy (21). Medication groups and a total number of regular home medications at admission were significantly associated with discrepancies and most common discrepancies were happened in Cardiovascular System and Nervous system drugs. This result was in contrast with research results (18), although other investigation showed same results (11). Nevertheless, the Cardiovascular System groups were not significantly correlated with discrepancies. Although the correlation between total number of regular home medications at admission and unintended medication discrepancy was anticipated because previous studies revealed this relationship (7, 22). In addition, our results showed, the high-risk drugs, have more potential to harm, were present in drug regimen of 56.5% patients and number of high-risk drugs variable were significantly associated with discrepancies. In addition, the number of unintended medication discrepancies in patient’s drug regimen containing high-risk drugs (88.5%) was higher than patient’s drug regimen that had not these drugs (11.5%) and with increasing of high-risk drugs in patient’s drug regimen the number of unintended medication discrepancies increased. Earlier investigations showed that 14.7% to 66.2% of unintended medication discrepancies at admission or discharge had potential harm to patients (23, 24). On the other hand, the 10% of unintended medication discrepancies had potential harm to patients (18). Pharmacist’s ability to obtain more exact and proper medication histories has been previously indicated (25); supervision absence of clinical pharmacists in the emergency department, lack of using medication reconciliation system and physicians attention to patients’ medication history may be the reasons of higher unintended medication discrepancies in patient’s drug regimen containing high-risk drugs.

In this cross-sectional study, population was mainly older patients (45.5%) that affected by discrepancies in the medication history at admission to hospital. Despite our result, higher age was a significant predicting element of discrepancies in some studies. Because of comorbidities, the number of prescribed medications increased with the increasing age (6, 12). “Well-designed processes for medication history verification were more important than patient characteristics” (5, 20). Our results showed that age and other demographical factors were not affected on discrepancies. However, the number patients in this study were lower than some other studies and our results might have differed if we studied more patients. We also did not find significant relationship between any chronic diseases that investigated in this study with discrepancies while patients with asthma were 6 times more likely to have unintended medication discrepancies (26). Like our results, there was not independent relationship between having a reconciliation error and suffering from diabetes mellitus or heart failure. The association between unintended discrepancies and chronic diseases has not been commonly studied.

## Conclusion

Medication discrepancies occur commonly on hospital emergency department in admission time and some medications that involved in these discrepancies could cause harm to patient. Understanding the type, frequency and type of discrepancies can help clinicians better understand ways to prevent them. In this regard, structured medication reconciliation process may help to prevent admission medication discrepancies and seem presence of hospital and clinical pharmacists in the emergency department increase the safety of patients in the admission. We suggested that implementation and using a computerized tool for detecting medication discrepancies could help simplify the process of medication reconciliation.

## Ethical considerations

Ethical issues (Including plagiarism, informed consent, misconduct, data fabrication and/or falsification, double publication and/or submission, redundancy, etc.) have been completely observed by the authors.
